# A Surprising Finding of Remote Ischial Avulsion

**DOI:** 10.5811/westjem.2015.5.27318

**Published:** 2015-10-20

**Authors:** Jill Tirabassi, Jessica Bull, Hannah M. Foley, Morteza Khodaee

**Affiliations:** University of Colorado School of Medicine, Department of Family Medicine, Denver, Colorado

A 25-year-old male presented to the ski clinic after colliding with a tree while snowboarding. He had immediate sharp pain at his “tailbone,” but denied numbness and weakness. Past medical history was initially reported as unremarkable. On exam, he demonstrated midline tenderness over the sacrum. Pelvic radiography was performed (Figure).

Imaging revealed an acute vertebral fracture, but it also demonstrated a large irregular left ischium to our surprise. When questioned further, the patient reported a hamstring injury in high school leading us to diagnose this finding as an old left ischial apophysial avulsion injury resulting in an osseous excrescence.

Ischial avulsion injuries are most commonly seen in adolescence and young adults. 1 Ischial avulsion injury tends to happen during a strong contraction of the hamstring muscles with activity like sprinting or jumping. 1–3 In puberty, the secondary ossification center appears at the apophysis and does not fuse until adolescence. 1 This bone is weak compared to muscle and ligaments; therefore, the young skeleton is more prone to fracture and osseous avulsions. 4–5

Radiograph is recommended to evaluate for a possible avulsion injury if there is pain over the ischial tuberosity, swelling at the hamstring origin or a palpable step-off. 1–2 Diagnosis is often made by plain radiography; typically as a sliver of bone displaced inferiorly and laterally from the ischium. 6 Ultrasound can be used to view other hamstring injuries; however, deep injuries often require magnetic resonance imaging, especially in athletes with massive muscle masses. 4–5

Avulsion fracture can commonly be misdiagnosed as a hamstring strain. However, unlike a muscular hamstring injury, avulsion fracture requires longer recovery, avoidance of hamstring stretching for four weeks and possible surgery. 1, 3 However, other forms of rehabilitation can be started at the time of diagnosis. The major indication for surgery is displacement of bone fragment greater than two centimeters. 2 If left untreated, the patient may experience recurrent discomfort with sitting for periods of time, pain with running, and even muscle wasting. 1 Also, the displaced fragment can lead to an exaggerated healing process and large mass of bone that can mimic neoplasm, such as an osteochondroma or even an Ewing’s sarcoma in the subacute phase of healing. 4–6

Underlying pathology that should be considered with ischial avulsion injuries include apophysitis of the tuberosity, bone tumor, metastases and osteoporosis. 5 Myositis ossificans traumatica is often seen following a sports injury, but is rarely seen in the hamstrings. 4

## Figures and Tables

**Figure:**
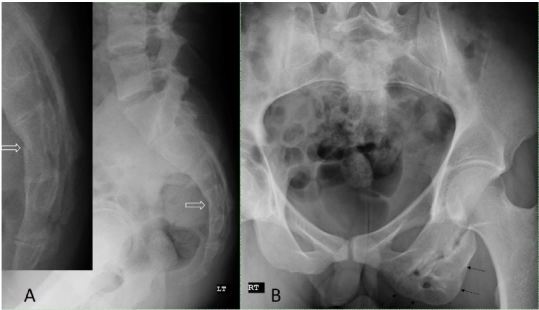
(A) Lateral pelvic radiograph demonstrates a minimally displaced transverse fracture of the S4 vertebrae (open arrows); (B) Anteroposterior pelvic radiograph reveals an incidental old left ischial apophysial avulsion injury (arrows).
